# Vibrio cholerae adhesin-derived peptides mediate strong pull-off forces in aqueous media with high ionic strength

**DOI:** 10.1016/j.colsurfb.2025.115390

**Published:** 2025-12-24

**Authors:** Syeda Tajin Ahmed, Sixin Zhai, Xin Huang, Sarvagya Sulaja, Adekunle Adewole, Alisa Ioffe, Andrea D. Merg, Jing Yan, Roberto C. Andresen Eguiluz

**Affiliations:** a Department of Chemical and Materials Engineering, University of California, Merced, Merced, CA 95344, USA; b Materials and Biomaterials Science and Engineering Graduate Group, University of California Merced, Merced, CA 95344, USA; c Department of Molecular, Cellular & Developmental Biology, Yale University, New Haven, CT 06511, USA; d Chemistry and Biochemistry Graduate Group, University of California Merced, Merced, CA 95344, USA; e Department of Chemistry and Biochemistry, University of California Merced, Merced, CA 95344, USA; f Health Sciences Research Institute, University of California Merced, Merced, CA 95344, USA; g Quantitative Biology Institute, Yale University, New Haven, CT 06511, USA

**Keywords:** wet adhesion, bacteria-inspired adhesion, Vibrio cholera, Surface Forces Apparatus

## Abstract

In this study, the pull-off forces of adsorbed films of four Bap1-inspired peptides in various solvents were investigated on negatively charged mica substrates using the surface forces apparatus (SFA), complemented with dynamic light scattering (DLS) for characterizing the aggregation behavior of peptides in solution. Bap1-inspired peptides consisted of the 57 amino acid wild-type sequence (WT); a scrambled version of the WT used to investigate the impact of the primary amino acid sequence in pull-off forces (Scr); a ten amino acid sequence rich in hydrophobic content (CP) of the WT sequence, and an eight amino acid sequence (Sh1) that corresponds to the pseudo-repeating sequence in the 57 AA. SFA results showed remarkable pull-off forces for CP, particularly in the presence of salts: measured pull-off forces were 26.0 ± 7.0 mN/m for no dwell-time and up to 42.0 ± 8.8 mN/m when surfaces were left in contact for 30 min. Sh1 did not bridge mica surfaces, while large aggregates clouded SFA measurements of Scr. DLS observations indicated that salts favored large peptide aggregation for all constructs (*D*_H_ > 1 μm), as compared to milliQ (*D*_H_ ≈ 100–500 nm) water and DMSO (*D*_H_ ≈ 5 nm), resulting in heterogeneous peptide film thicknesses.

Adhesion in the presence of water poses significant challenges because water interferes with adhesive-substrate interactions. These challenges include weakened electrostatic interactions due to ion screening [1], competition with water molecules and hydration layers [2], reduced van der Waals interactions [3], or oxidation [4]. Understanding how to overcome these challenges is crucial for developing wet and salt-tolerant materials in wet environments, such as biomedical glues, marine infrastructure sealants, and a molecular understanding of biofilm formation for developing peptide-based antibacterial agents. Organisms, such as bacteria, have developed strategies to overcome challenges in the presence of water. For example, bacterial colonies rapidly cover any available surface via specific and nonspecific interactions in benthic environments or the human body. To do so, bacteria express multiple adhesive biomolecules, such as siderophores [5] and adhesins [6] to interact with its environment and colonize surfaces efficiently. Recently, a 57-amino-acid (AA) sequence in the Bap1 adhesin in the biofilm of *Vibrio cholerae* (Fig. 1a), the causal agent of the pandemic cholera, has been identified as a critical adhesion mediator to multiple abiotic and biotic surfaces in *V. cholerae* biofilm, such as lipids and silica surfaces, respectively [7,8]. This peptide, rich in hydrophobic and positively charged amino acids, has the potential to be an essential “blueprint” provided by nature for identifying new adhesive motifs based solely on canonical amino acids.

Multiple organisms have inspired research into developing wet adhesion technologies. Snails, for example, secrete various types of mucus to facilitate adhesion [11-13]. Studies of tick saliva or cement, which contain glycine-rich proteins chaperoned by cationic and aromatic amino acids, are yet another example of efficient and robust wet adhesion [14], rich in moieties that drive π-π and cation-π interactions [15]. Indeed, π-π and π-cation interactions, and complementary intra- and intermolecular forces, such as hydrogen bonding, are essential, enabling strong wet cohesion and adhesion [16] in several marine organisms [14,17-19]. Many of these findings have originated from marine mollusks, such as mussels and barnacles. These are the organisms that have unleashed the most significant wave of wet adhesion technology promises [20-28]. That is in part due to the abundance of catechol present in the adhesive proteins in the form of dihydroxyphenylalanine (DOPA, a posttranslational modification of tyrosine). However, given the reactivity of the catechol moiety of DOPA [29,30], making it more susceptible to degradation to environmental factors, other catechol-inspired, more stable moieties have been explored as potential adhesion mediators [31,32].

In this study, the pull-off forces, as key measurements of surface interactions, of physically adsorbed films of four Bap1-inspired peptides in various solvents, were investigated on negatively charged mica substrates using the surface forces apparatus (SFA), using a symmetrical configuration of back-silvered micas (droplet experiment, at 24 °C, water in the chamber to minimize droplet evaporation, and surfaces with a radius of curvature of 2 cm). SFA measurements were complemented with dynamic light scattering (DLS) to characterize the aggregation behavior of peptides in solution. Experimental details are provided in the SI Materials and Methods sections.

Bap1-inspired peptides consisted of the 57 amino acid wild-type sequence (WT); a scrambled version of the WT (abbreviated as Scr) used to investigate the impact of the primary amino acid sequence in pull-off forces; a shorter ten amino acid sequence rich in hydrophobic content corresponding to the central portion (CP) of the WT sequence that was recently identified to be critical for lipid binding [33]; and an eight amino acid sequence named short-1 (Sh1) that corresponds to the pseudo-repeating sequence (appearing 4 times in WT and bolded in Table 1) in the 57 AA (Fig. 1b, and SI S1 Bap1-inspired peptide details), all synthesized from at least two independent sources (SI S2 Bap1-peptide synthesis and characterization). Peptides were dissolved in dimethyl sulfoxide (DMSO) at a stock concentration of 150 μM and used to prepare working solutions of 1.5 μM of peptides in either aqueous solvents with high ionic strength (M9 buffer at pH 7, with a total ionic strength of 190 mM, which is a common bacteria growth medium) or in deionized water (milliQ water at pH 7, no salts), or further diluted with an organic solvent (pure DMSO) to 1.5 μM. To quantify the pull-off forces of WT, Scr, CP, and Sh1 peptide films deposited onto mica substrates from an M9 aqueous buffer (Fig. 2a), the SFA was used. SFA results showed remarkable bridging forces (pull-off forces) for CP, particularly in the presence of ions (M9): measured pull-off forces were 26 ± 7 mN/m (6 ± 1 mJ/m^2^) for no dwell time and up to 42 ± 9 mN/m (9 ± 2 mJ/m^2^) when surfaces were left in contact for 30 min. The increased pull-off forces observed with longer contact times could be due to peptide rearrangement. DLS observations indicated that ions present in M9 favored large peptide aggregation for all constructs (with hydrodynamic diameter, *D*_H_ > 1 μm), as compared to milliQ (*D*_H_ ≈ 100–500 nm) water and DMSO (*D*_H_ ≈ 5 nm), resulting in heterogeneous peptide film thicknesses, as measured by SFA. This study concludes with a comparison to the pull-off forces of mussel foot protein (Mfps) inspired peptides reported in the literature.

Representative SFA force-distance curves in M9 buffer, measured for WT and CP are shown in Fig. 2b. Sh1 exhibited purely repulsive forces with no pull-off forces measured. This could be due to the absence of surface-interacting units in the sequence (but present in CP), particularly phenylalanine, which has been reported to enhance cohesion in DOPA-containing underwater adhesives [34]. Scr formed large aggregates, resulting in double contacts, and it was not possible to extract pull-off forces from the force spectroscopy measurements performed with SFA. This finding underscores the importance of the primary structure over amino acid composition in determining functionality. All force-distance curves for WT and CP exhibited purely repulsive forces during approach, followed by an abrupt jump out of contact during retraction of the surfaces, which was distinct in magnitude from that observed for the M9 buffer alone (Figure S2). Sh1 did adsorb to the mica, forming a peptide film, but only repulsive forces were detected during approach, and no jump-out of contacts occurred. Pull-off forces (force normalized by radius of curvature required to separate the two participating surfaces), -*F/R*, for WT and CP peptides were measured to be 18 ± 1 mN/m and 26 ± 7 mN/m, respectively, with no dwell time (total contact time of approximately 2 min, the time it takes to complete the loading/unloading cycle). Leaving the surfaces under the maximum applied load (~100 mN/m) for an additional 10 min or 30 min had a marginal effect on WT films, increasing the pull-off forces to 21 ± 2 mN/m (Fig. 2c). For CP, however, pull-off forces increased to 29 ± 9 mN/m and 42 ± 9 mN/m for 10 and 30 min, respectively, corresponding to an increase of 10 % and 60 % in the magnitude of pull-off forces. These observations suggested that most of the intra- and intermolecular interactions responsible for the adhesive and cohesive properties of the WT nanofilm were formed during the shorter contact times and remained stable. For CP, however, intra- and intermolecular interactions responsible for the adhesive and cohesive properties of the CP nanofilm appeared to increase, potentially due to a higher degree of conformational flexibility of the nanofilm and providing a higher density of effective “sticky” moieties per peptide molecule, or differences in film morphologies. This was further evidenced by the different film thicknesses observed between WT and Sh1, and between CP and WT, with CP forming significantly thicker nanofilms (Fig. 2d). Details on the SFA and the symmetric peptide deposition procedures can be found in the SI sections S4 Surface Forces Apparatus and substrate preparation and S5 Peptide film deposition. Based on the differences in aggregate sizes in bulk (DLS) and onset of interactions as measured with the SFA, these aggregates may dissociate upon surface adsorption, albeit non-homogeneously. However, more research is needed to pinpoint.

It is well established that salts affect intra- and intermolecular forces, such as cation-π or hydrogen bonds [35-37]. To investigate the adhesive and cohesive performance of the peptide films in the absence of salts, WT, Scr, CP, and Sh1 peptide films were deposited onto mica substrates from milliQ water, yielding an experimental configuration similar to that used for M9 buffer measurements (Fig. 2a). Representative SFA force-distance curves in milliQ water, measured for WT, CP, and Sh1 are shown in Fig. 3a. All force-distance curves for WT, Scr, and CP, exhibited purely repulsive forces during approach. Scr formed large aggregates, resulting in double contacts, and it was not possible to extract F-D profiles of the force-spectroscopy measurements performed with the SFA, similar to what was observed for Scr in M9. Only CP had an abrupt jump out of contact during retraction of the surfaces. The pull-off forces, -*F/R*, for CP in milliQ water were 10 ± 3 mN/m, with no dwell time. Leaving the surfaces under the maximum applied load (~100 mN/m) for an additional 10 min or 30 min had no impact on CP film pull-off forces, remaining at 10 ± 4 mN/m and 10 ± 5 mN/m, respectively (Fig. 3c). Similar to the M9 case, these observations suggested that intra- and intermolecular interactions responsible for the adhesive and cohesive properties of the CP nanofilm were established during the shorter contact times and increased with increasing contact times. Contrary to what was measured in the M9 buffer, WT and Sh1 peptide nanofilms in milliQ water were thicker than CP (Fig. 3c) and larger than those formed from the M9 buffer, despite the negligible pull-off forces, suggesting that physical absorption is not the only factor determining the pull-off force.

Lastly, we assessed the pull-off forces of WT, Scr, CP, and Sh1 peptides dissolved in DMSO, an organic solvent commonly used to solvate hydrophobic peptides, such as those investigated in this study. A droplet at a bulk concentration of 1.5 μM of each peptide was deposited in a symmetric configuration, similar to the configuration used for M9 and milliQ water (Fig. 2a). Purely repulsive forces were measured for WT, CP, and Sh1 during loading (approach), and no jump-outs of contact during retraction were detected (Figure S3). For the Scr peptide, no pull-off forces were collected due to the consistently large aggregates resulting in double contacts and long-range repulsion.

We now put our results into the context of existing literature on underwater adhesives, particularly those based on Mfps. Mfp-3 and Mfp-5, two of the Mfps with the highest catechol content, have 21 % and 27 % dihydroxyphenylalanine (DOPA) [38,39]. Higher contents of DOPA, as tested in synthetic constructs, such as PEGtides [35] and peptides [34], did not increase the wet adhesion performance, indicative that other intra- and intermolecular interactions synergize for optimal surface bridging. The *V. cholerae* Bap1-inspired peptides presented here, specifically WT (*F*/*R* ~ 18 mN/m) and CP (*F*/*R* ~ 25 mN/m), compared to Mfp-3 (*F*/*R* ~ 2–3 mN/m) [40] and Mfp-5 (*F*/*R* ~ 7–8 mN/m) [41], showed higher pull-off forces under similar experimental conditions. That is, similar bulk molarities during peptide deposition (1.5–2.5 μM), similar ionic strengths (100 mM or higher), dwell times (2 min), and mica as the model substrate, using an SFA. However, key differences with probable performance impact are pH values and salt compositions. We also note that the pull-off forces of CP nanofilms could be further improved by increasing the dwell time. While mechanistic details are yet to be elucidated, backbone rigidity and peptide reorientation could lead to the increased pull-off forces with increased dwell time. Similar time-dependent pull-off forces have been reported for Mfps [40,41]. Furthermore, the peptides used in this study consisted exclusively of canonical amino acids (no DOPA), offering advantages such as ease of synthesis and computational modeling, reduced chemical and structural complexity, and stability, among others. The performance of CP is partially attributed to its composition: 50 % aromatic-rich amino acids, 40 % content of polar amino acids, and 10 % cationic content (Fig. 1b). Based on back-of-the-envelope calculations, each CP peptide could form a maximum of 10 possible π-π bonds, two possible cation-π interactions, and five hydrogen bonds (donors or acceptors) with a surface or another peptide. Details on the estimates can be found in *S8 Estimation of intermolecular interactions*. The final, optimal number will depend on the peptide's conformational flexibility, side-chain orientation, and environmental factors, such as pH and the nature of the ions present, as in the case of the M9 buffer, which is rich in phosphates. However, this estimate should be considered very rough and may be overestimating the number of interactions, given the system's complexity. On the other hand, sequence also matters: the WT showed considerable pull-off forces, while the Scr version with the same composition had no measurable force. The contribution of the secondary structure of the peptide to adhesion will be an interesting topic for future study. Indeed, recently we observed an interesting conformational change of WT in contact with lipid bilayers [33], but we do not yet have such information for abiotic surfaces.

A challenge encountered was the frequent presence of large aggregates that made SFA measurements difficult. On average, one out of three independent experiments (one independent experiment consisted of a freshly prepared pair of mica substrates) was successful, which is not uncommon for SFA experiments. To quantify aggregate sizes, dynamic light scattering (DLS) was used, and confirmed the presence of large aggregates (Figure S4 and S9. Peptide hydrodynamic size measured via dynamic light scattering), with estimated *D*_H_ for WT and CP aggregates larger than 1 μm when dissolved in M9 buffer, decreasing to hundreds of nm when dissolved in milliQ water. This is not surprising, as, in addition to the rich hydrophobic content, the presence of diphenylalanine (FF) in the sequence of the WT and CP was likely an additional driver for self-assembly, known to participate in β-amyloid-like fibril formation [42]. It is, however, necessary to use other characterization approaches in future studies, such as atomic force microscopy (AFM) and quartz crystal microbalance with dissipation (QCM-D), to confirm trends and expand mechanistic understanding.

In summary, the underwater pull-off forces of *V. cholerae-inspired* peptides were studied using the SFA. For the first time, direct force spectroscopy of Bap1-peptide sequences that mediate biofilm adhesion and derivatives was investigated to identify new molecular motifs, based on canonical amino acids, that will aid in determining molecular design rules for robust underwater adhesives at neutral pH and in high-ionic-strength environments. The CP peptides exhibited excellent cohesive energy, reaching approximately 8 mJ/m^2^ in M9 buffer and approximately 4.5 mJ/m^2^ in milliQ water. Our results emphasize the importance of π-π stacking and cation-π, in addition to hydrogen bonding, for the design of wet adhesives for potential use in marine, biomedical, and antifouling applications. Open questions remain, and how these *V. cholerae*-inspired peptides perform across a more expansive parameter space, such as surface chemistry and the impact of kosmotropes or chaotropes on film morphologies, should be more systematically explored in the future.

## Supplementary Material

1

## Figures and Tables

**Fig. 1.**
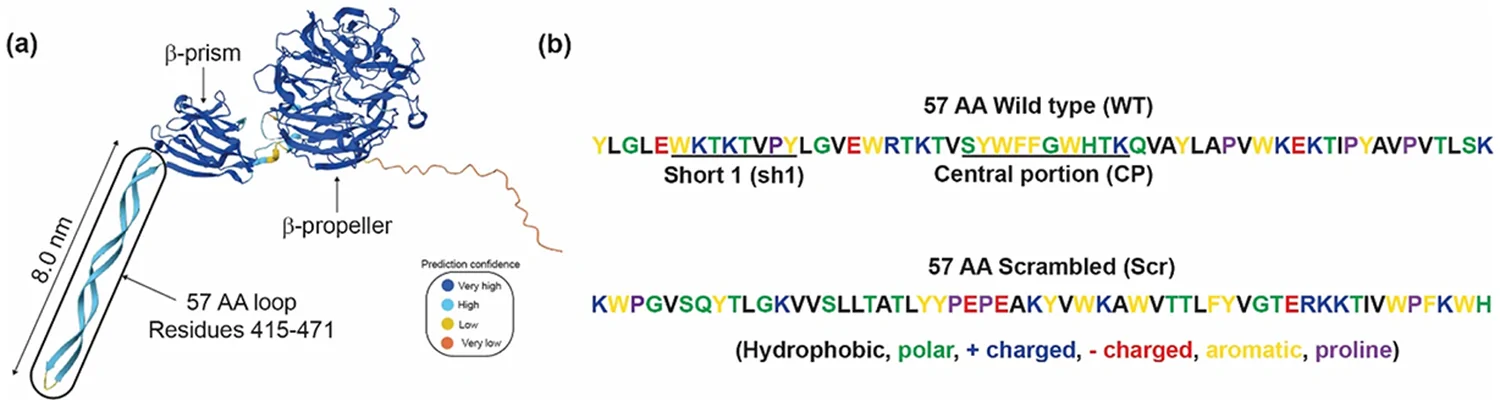
(a) 3-D representation of the *V. cholerae* adhesin Bap1, showing the main β-propeller domain, the β-prism, and the 57 AA loop relevant to this study. The β-propeller and β-prism domains have been crystallized [7], where the 57 AA loop structure comes from prediction of AlphaFold [9,10]. (b) The color-coded 57 AA sequence of the wildtype (WT) loop shown in (a), and two additional peptides investigated in this study, short 1 (Sh1) and central portion (CP), as well as a scrambled (Scr) analog of the WT 57 AA.

**Fig. 2.**
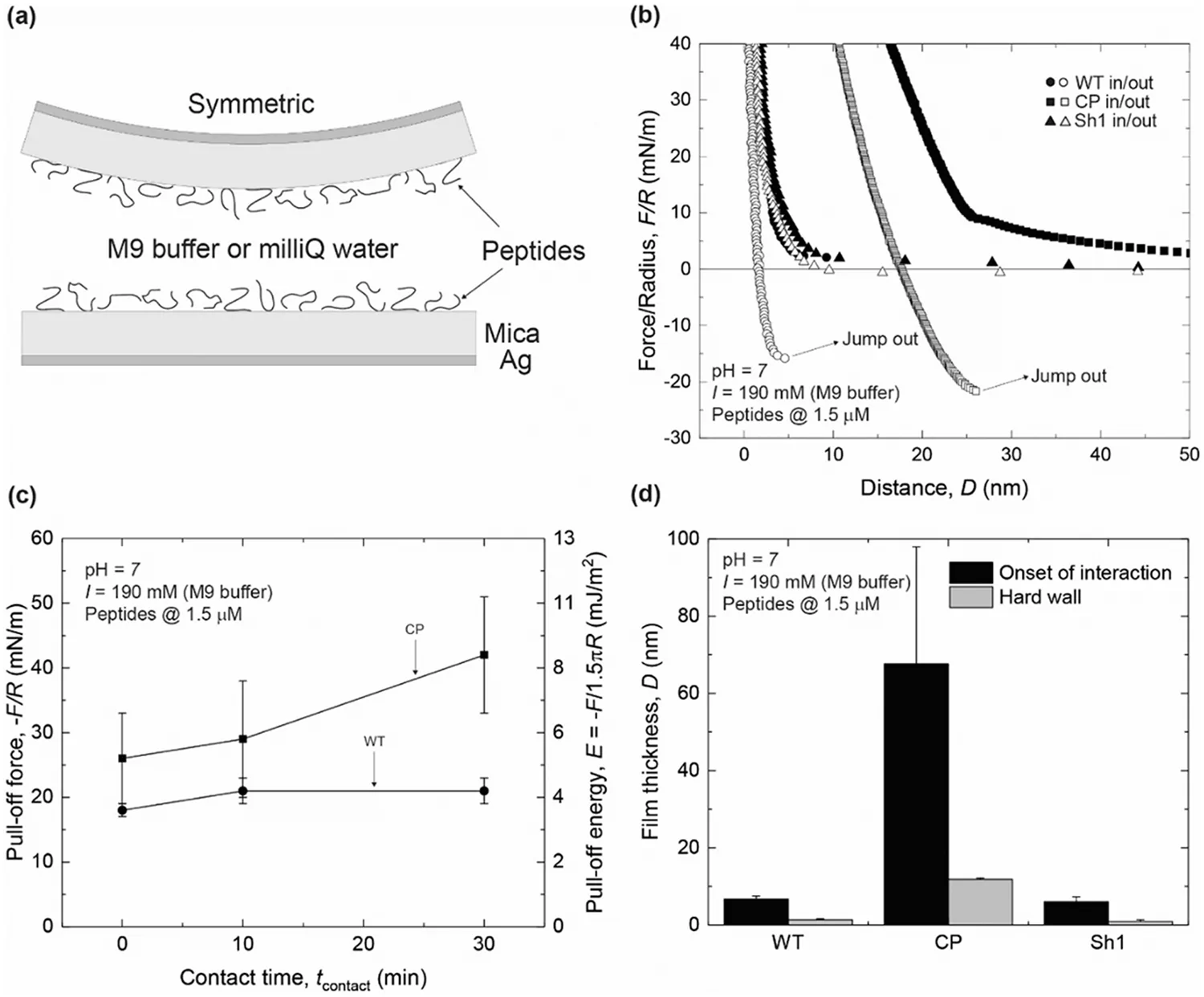
(a) SFA symmetric configuration used in these studies, consisting of back-silvered mica as substrates. Each mica supported a Bap1–57AA nanofilm deposited from a droplet of 40 μL at 1.5 μM, bridging the two surfaces. (b) Representative force-distance curves obtained during a loading and unloading cycle of WT (circles), CP (squares), and Sh1 (triangles) in M9 buffer. (c) Plots of pull-off forces normalized by radius of curvature, -*F/R*, versus contact time, *t*_contact_, for WT and CP peptides in M9 buffer. (d) Absolute film thicknesses at rest (onset of interaction) and at maximum compression (hard wall). Film thickness is reported as the thickness of the two films shown in the schematic (a). For CP, *N* = 2 and *n* = 27, 7, 7; for WT, *N* = 2 and *n* = 30, 5, 5 for *t*_contact_ = 0, 10, and 30 min, respectively.

**Fig. 3.**
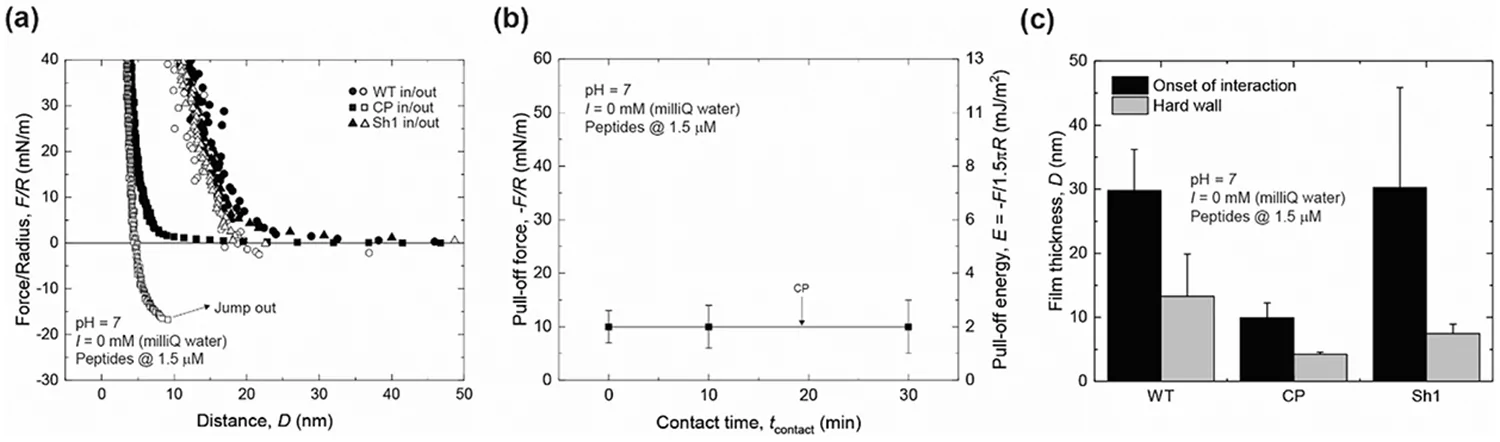
(a) SFA symmetric configuration representative force-distance curves obtained during a loading and unloading cycle of WT (circles), CP (squares), and Sh1 (triangles) in milliQ water. (b) Plots of pull-off forces normalized by radius of curvature, -*F/R*, vs contact time, *t*_contact_, for CP peptides in milliQ water. Film thickness is reported as the total thickness of compressed peptide films when the two surfaces approach, as shown in the schematic of Fig. 2(a). For CP, *N* = 3 and *n* = 32, 7, 8; for WT, *N* = 2 and *n* = 24, 0, 0 for *t*_contact_ = 0, 10, and 30 min, respectively.

**Table 1:** Library of Bap1-inspired peptides. Bolded are the pseudo-repeating unit of WT synthesized as Sh1.

Peptide	Sequence	Molecular weight, MW (Da)	Solvents tested
WT	YLGLE**WKTKTVPY**LGVE**WRTKTVSY**WFFG**WHTKQVAY**LAPV**WEKTIPY**AVPVTLSK	6793	Water, M9, DMSO
Scr	KWPGVSQYTLGKVVSLLTATLYYPEPEAKYVWKAWVTTLFYVGTERKKTIVWPFKWH	6793	Water, M9, DMSO
Sh1	**WKTKTVPY**	1022	Water, M9, DMSO
CP	SYWFFGWHTK	1359	Water, M9, DMSO

## Data Availability

Data will be made available on request.

## Appendix A. Supporting information

Supplementary data associated with this article can be found in the online version at doi:10.1016/j.colsurfb.2025.115390

## Abbreviations:

SFA: surface forces apparatus
Mfps: mussel foot proteins
WT: wild type
Scr: scrambled
Sh1: short 1
CP: central portion
DOPA: dihydroxyphenylalanine.
